# Modeling multi-sensory feedback control of zebrafish in a flow

**DOI:** 10.1371/journal.pcbi.1008644

**Published:** 2021-01-22

**Authors:** Daniel A. Burbano-L., Maurizio Porfiri

**Affiliations:** 1 Department of Mechanical and Aerospace Engineering, Tandon School of Engineering, New York University, New York City, New York, USA; 2 Department of Biomedical Engineering, Tandon School of Engineering, New York University, New York City, New York, USA; 3 Center for Urban Sciences and Progress, Tandon School of Engineering, New York University, New York City, New York, USA; University of Pittsburgh, UNITED STATES

## Abstract

Understanding how animals navigate complex environments is a fundamental challenge in biology and a source of inspiration for the design of autonomous systems in engineering. Animal orientation and navigation is a complex process that integrates multiple senses, whose function and contribution are yet to be fully clarified. Here, we propose a data-driven mathematical model of adult zebrafish engaging in counter-flow swimming, an innate behavior known as rheotaxis. Zebrafish locomotion in a two-dimensional fluid flow is described within the finite-dipole model, which consists of a pair of vortices separated by a constant distance. The strength of these vortices is adjusted in real time by the fish to afford orientation and navigation control, in response to of the multi-sensory input from vision, lateral line, and touch. Model parameters for the resulting stochastic differential equations are calibrated through a series of experiments, in which zebrafish swam in a water channel under different illumination conditions. The accuracy of the model is validated through the study of a series of measures of rheotactic behavior, contrasting results of real and *in-silico* experiments. Our results point at a critical role of hydromechanical feedback during rheotaxis, in the form of a gradient-following strategy.

## Author summary

The astounding feats of animal orientation and navigation have fascinated scientists and engineers for decades. The refined and elegant processes of orientation and navigation are generally thought to be the outcome of a complex feedback process, which involves the integration of multiple cues gathered from the surroundings. Fish rheotaxis is an innate behavior through which an animal is able to orient itself and swim against a current, even in the absence of visual cues. To date, little is known about the information pathways that underlie this behavior and how they are integrated. To help address this challenge, we propose a data-driven mathematical model of rheotaxis in zebrafish— an emerging species of choice in biomedical research. Our model explains how zebrafish make use of visual, hydrodynamic, and tactile cues in a feedback loop to adjust their heading and speed during swimming. We validate the accuracy of our model by comparing real and synthetic data across two experimental conditions, in which we vary the illumination of a water channel. Our results demonstrate how a simple, yet effective, feedback control mechanism can explain a complex process such as rheotaxis.

## Introduction

The ability of animals to orient themselves and navigate in complex environments has fascinated scientists and engineers for decades [1–3]. Understanding the mechanisms underlying this behavior is of paramount importance in behavioral ecology for elucidating complex processes such as foraging [4], mating [5], and survival [6]. Animal orientation and navigation has also inspired technological solutions ranging from sensors [7] to computer algorithms for coordinating teams of construction robots [8].

Animal orientation and navigation typically involves the integration of different sensory systems such as vision, olfaction, and touch. These systems are used to gather information from the surrounding environment, which is, in turn, used to “close the loop” by the animal. Using this information, the animal can adjust its position and orientation. Remarkable examples include homing in salmons, which use a combination of geomagnetic and olfactory cues to swim back to their natural streams to spawn, after spending several years in the open ocean [9, 10]. Moths, on the other hand, are able to use intermittent olfactory cues in odor plumes to control their maneuvers to reach their mating partner [11]. Interestingly, navigation and orientation can be very complex even for insects, which are far in the evolutionary tree from vertebrates [12].

In some cases, animals display a specific orientation of locomotory behavior (taxis), elicited by environmental stimuli like gravity (geotaxis) [13], light (phototaxis) [14], or fluid flow (rheotaxis). For instance, fish rheotaxis is an innate behavior from early stages of life [15] that is essential for survival [16–18]. This behavior can be performed even in the absence of visual cues [19], whereby fish can use their lateral line to aid their navigation in the dark [17, 20]. The lateral line consists of a collection of neuromasts (clusters of sensory cells), sensitive to changes of water pressure, that enable a fish to create a hydrodynamic image of the surroundings [21–23]. Empirical evidence suggests that the lateral line plays a key role in the animals’ orientation process [15, 20, 24]. For example, it has been recently shown that larval zebrafish use the lateral line to estimate the local vorticity of the surrounding fluid flow, which aids their orientation process [25].

In general, rheotaxis is regarded as a multi-sensory feedback process that integrates visual, hydromechanical, olfactory, and even tactile cues [26, 27]. A full understanding of how all the sensory information is processed by rheotacting fish is yet to be established. Here, we seek to contribute insight into the mechanisms underlying rheotaxis through the development of a data-driven mathematical model of adult zebrafish locomotion in a fluid flow.

Zebrafish is a freshwater species, which has been widely used as a model organism for its several advantages, ranging from its fully-sequenced genome to physiological and neurological homologies with humans [28, 29]. Zebrafish have been used in a wide array of preclinical efforts, from drug discovery [30] to the study of complex brain disorders such as depression, autism, and psychoses [31]. The possibility of investigating the neural and genetic basis of behavior through zebrafish [32] offers compelling motivation for the study of their rheotactic response.

Mathematical models of zebrafish locomotion have been shown to be a powerful tool to complement and inform experimental research. For instance, in [33], a simple mathematical model of the burst-and-coast swimming style of zebrafish revealed that adult fish have longer coasting due to their larger body mass and higher speed at the beginning of a burst. Data-driven models of fish locomotion typically describe the time evolution of the heading and the linear speed of fish using stochastic differential equations (SDEs) [34–38]. For instance, a pair of coupled Ornstein-Uhlenbeck processes were proposed in [36] to model the coupled evolution of the turn rate and speed of adult zebrafish. Similarly, the jump persistent turning walker was introduced to faithfully capture the burst-and-coast swimming of zebrafish in two [37] and three dimensions [39]. Building on these efforts, mathematical models have addressed the role of spatial constraints on zebrafish range of vision [40], as well as pharmacological manipulations [41, 42].

Common to this entire body of literature on mathematical modeling of zebrafish locomotion is the premise of a quiescent fluid environment. In its natural habitat, however, zebrafish can experience different flow speeds between 3.5 to 13.9 cm/s [43]. Recent experimental research points to the critical role of water flow on the collective response of zebrafish [44]. Existing mathematical models largely exclude the effects of a fluid flow, thereby challenging the study of rheotaxis. To the best of our knowledge, the only mathematical models of fish rheotaxis in literature are the phenomenological model proposed by [45] and the kinematic model by [46]. In [45], the authors established a minimalistic model of rheotaxis based on a Kuramoto-like oscillator, which describes fish heading through a bias towards the flow source. Similarly, in [46], the authors proposed a kinematic model with a bias towards the flow in the form of a linear feedback of the differential pressure sensed by the animal. Despite their promise, these models neither consider the flow physics nor the multi-sensory feedback that fish should employ to orient and swim in the flow.

A potential approach to develop a data-driven model of zebrafish rheotaxis is to leverage recent theoretical results on finite-dipole models of animal swimming [47, 48]. Within the finite-dipole model, a fish is assimilated to a pair of point vortices separated by a finite distance [47], whose strengths can adapt according to behavioral rules [48]. Based on this modeling paradigm, we explore a multi-sensory feedback control system, which allows the animal to adjust its orientation as a function of visual, hydromechanical, and tactile cues.

More specifically, we expand on the finite-dipole paradigm to encompass a data-driven model that allows the fish to adjust the vortex strengths as a function of multi-sensory input from the surroundings. Sensory input from the lateral line is used to estimate the local circulation of the fluid flow, and visual and tactile cues inform the interaction with the walls. The model is calibrated using a data set consisting of overhead recordings of adult zebrafish swimming in a water channel in standard illumination conditions or in the dark. We demonstrate the effectiveness of our approach by comparing the scoring of behavioral metrics on real and synthetic data from *in-silico* experiments.

## Results

### Experiments

We conducted 24 experiments where adult zebrafish individually swam in the flow. In order to understand the role of vision in the fish swimming mechanism, we considered two experimental conditions on groups of 12 individuals: Bright and Dark. In Bright, fish swam with standard illumination (250 lx), and in Dark they swam in the darkness.

Based on related studies, we anticipated that vision would play an important role on rheotaxis [20, 26]. In particular, we expected zebrafish to reduce rheotactic behavior in the darkness when compared to standard illumination conditions [20, 25, 27]. In addition, we anticipated that, even when deprived from vision, fish would still be able to engage in counter flow swimming. In fact, touch and hydromechanical cues along with information from the vestibular system could be integrated to perform rheotaxis [17, 27]. In summary, we made the following hypotheses: (i) zebrafish would still be able to perform rheotaxis in the absence of visual cues, and (ii) the lack of illumination would decrease the ability of zebrafish to perform rheotaxis.

Using an automatic tracking software, we obtained time series for the position of the fish centroid and heading angle as described in Materials and methods. To quantify rheotaxis, we compared the scoring of two different metrics, the mean of (negative) cosine of the heading and the mean rheotaxis index (*RI*); see Materials and methods for a mathematical definition. Both metrics take values between −1 and 1 corresponding to biased headings towards downstream and upstream, respectively. A zero value represents the case in which a fish does not have a preference to swim either upstream or downstream.

**Fig 1:**
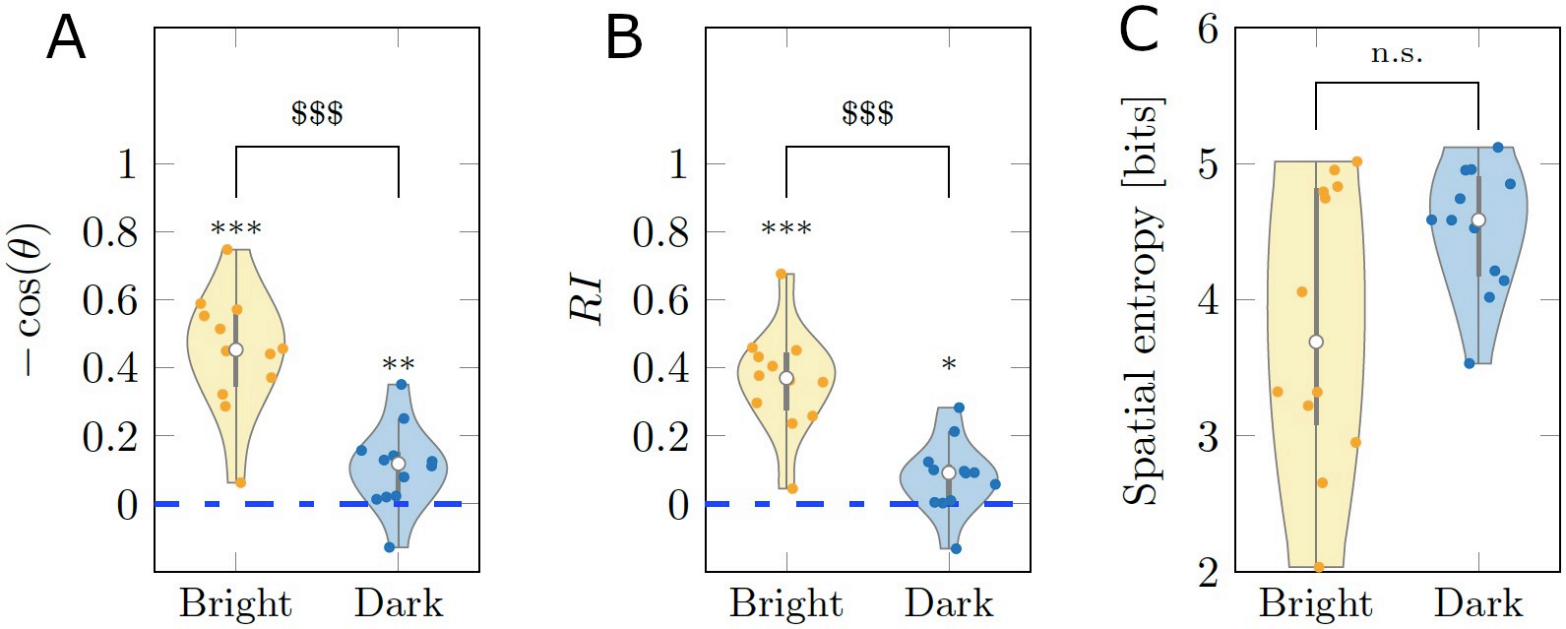
Average scoring of three behavioral metrics for real experiments. (A-B) Rheotactic metrics taking values between −1 and 1 corresponding to biased headings downstream and upstream, respectively. (C) Spatial entropy, measuring the exploratory behavior across the test section. The average was taken over the entire time series of 9, 000 points for each experimental subject. The gray bar in each violin plot details median (white dot), first and third quartiles, and lower and upper adjacent values. The colored area of a violin plot corresponds to the probability density of the data. Symbols *, ** and *** indicate significant differences from zero with *p* < 0.050, *p* < 0.010, and *p* < 0.001, respectively. Symbol $$$ indicates significant difference between conditions with *p* < 0.001.

From the results in Fig 1, we determined that the cosine of the heading was different from chance in both Bright (*V* = 78;*p* < 0.001) and Dark (*V* = 71;*p* < 0.010). Likewise, for *RI*, we registered significant differences from chance in both Bright (*V* = 78;*p* < 0.001) and Dark (*V* = 68;*p* = 0.050). These findings support the first hypothesis that fish can perform rheotaxis independent of the illumination conditions. In agreement with the second hypothesis, pairwise comparisons between Bright and Dark identified a superior rheotactic response for animals swimming in standard illumination conditions, with respect to the cosine of the heading (*W* = 134;*p* < 0.001) and *RI* (*W* = 134;*p* < 0.001).

Finally, to measure locomotory activity of the animal in the form of exploration of the entire test section, we calculated the spatial entropy; see Materials and methods for a mathematical definition. The comparison between the two conditions suggests the presence of a weak trend, with fish swimming in the dark displaying a higher locomotory activity than subjects swimming in standard illumination conditions (*W* = 47;*p* = 0.160). This weak trend was accompanied by a significant difference of the variance of the spatial entropy between conditions (*F* = 13.497;*p* < 0.010), with animals swimming in the dark displaying a lower variability.

### Zebrafish swimming as a finite-dipole

**Fig 2:**
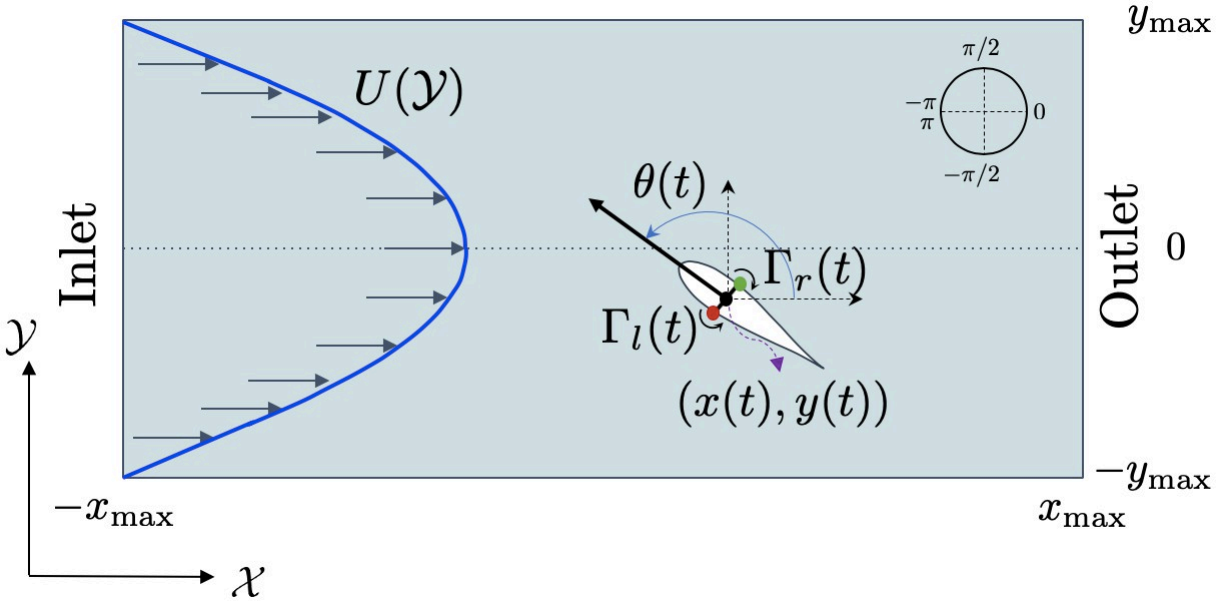
Modeling zebrafish swimming in a flow as a finite-dipole. U(Y) is the profile of the uniaxial background flow. The black dot and arrow denote the fish centroid position (*x*(*t*), *y*(*t*)) and heading angle *θ*(*t*), with respect to the global reference frame (X,Y). The green and red dots represent the left and right location of the vortices of circulation strengths *Γ*_*l*_(*t*) and *Γ*_*r*_(*t*), respectively.

We treat a zebrafish as a self-propelled body swimming in two dimensions within a uniaxial inviscid flow (see Fig 2). Here, (*x*(*t*), *y*(*t*)) are the coordinates of the fish centroid in the global reference frame (X,Y), where *t* is the time variable. The angle *θ*(*t*) ∈ [−*π*, *π*) represents the fish heading. For *θ* = −*π* the fish is heading upstream, while for *θ* = 0 it is heading downstream. Following [47, 48], we assimilate the fish to a finite-dipole, consisting of a pair of point vortices separated by a distance *l*, corresponding to the fish thickness. These two point vortices of circulation strengths *Γ*_*l*_(*t*) and *Γ*_*r*_(*t*) describe the fish self-induced propulsion. The fish thickness is about 5mm for adult zebrafish, which is much smaller than either dimensions of the water channel, 2*x*_max_ and 2*y*_max_.

Hence, the time evolution of the fish position and heading angle can be described by the following set of ODEs:

$$\begin{array}{c} \frac{dx(t)}{dt}=\frac{\Gamma_{l}\left(t\right)+\Gamma_{r}\left(t\right)}{4\pi l}\cos\left(\theta \left(t\right)\right)+U\left(y\left(t\right)\right), \end{array}$$ (1a)

$$\begin{array}{c} \frac{dy(t)}{dt}=\frac{\Gamma_{l}\left(t\right)+\Gamma_{r}\left(t\right)}{4\pi l}\sin\left(\theta \left(t\right)\right), \end{array}$$ (1b)

$$\begin{array}{c} \frac{d\theta (t)}{dt}=-U^{\prime }\left(y\left(t\right)\right)\cos^{2}\left(\theta \left(t\right)\right)+\frac{\Gamma_{l}\left(t\right)-\Gamma_{r}\left(t\right)}{2\pi l^{2}}, \end{array}$$ (1c)

where U(Y) and $U^{\prime }\left(Y\right)$ are the axial flow velocity and its gradient along the width of the channel, respectively; see Materials and methods for details on the derivation. These scalar spatial fields entirely capture the effect of the background flow on the fish motion.

The vortex strengths encapsulate the self-propelling mechanism along with the feedback contributions for controlling both heading and speed. In particular, Γ_*l*_(*t*)>Γ_*r*_(*t*) indicates that the fish performs a counterclockwise turn, while the opposite, Γ_*r*_(*t*)>Γ_*l*_(*t*), refers to clockwise turns. For Γ_*l*_(*t*) = Γ_*r*_(*t*), the fish swims straight. The fish relative speed with respect to the background flow is (Γ_*l*_(*t*) + Γ_*r*_(*t*))/(4*πl*).

### Modeling the time evolution of the vortex strengths

In general, the distributions of Γ_*l*_ and Γ_*r*_ are highly correlated suggesting that these processes are not independent. In particular, the processes unfold around the line Γ_*l*_ = Γ_*r*_ with random fluctuations corresponding to turning maneuvers. This behavior shares similarities with phase plots of diffusively coupled dynamical systems, often studied in the context of synchronization [49–51]. Just as oscillators tend to synchronize their phase against noise [52], the two vortices seek to match their circulation strengths against random fluctuations (see Supporting information S1 Fig).

We approximate the vortex strengths Γ_*l*_(*t*) and Γ_*r*_(*t*) by a Gamma distribution [53]. Based on the analogy with diffusively coupled systems, we propose the following pair of coupled Cox–Ingersoll–Ross processes [54] to model the time evolution of the vortex strengths:

$$\begin{array}{c} d\Gamma_{l}\left(t\right)=(\alpha (\beta -\Gamma_{l}\left(t\right))+u\left(t\right))dt+\sigma \sqrt{\Gamma_{l}\left(t\right)}dW_{l}\left(t\right), \end{array}$$ (2a)

$$\begin{array}{c} d\Gamma_{r}\left(t\right)=(\alpha (\beta -\Gamma_{r}\left(t\right))-u\left(t\right))dt+\sigma \sqrt{\Gamma_{r}\left(t\right)}dW_{r}\left(t\right), \end{array}$$ (2b)

where *α* [1/s] and *β* [cm^2^/s] are positive parameters representing the linear rate of decay and a baseline value of the vortex strengths, respectively. The parameter *β* is associated with the speed of the fish relative to the background flow, whereby *β*/(2*πl*) would be the relative speed of the finite-dipole during straight swimming, without the effect of noise. The positive parameter *σ* [cm/s] measures the strength of both added noises *W*_*l*_(*t*) and *W*_*r*_(*t*), which are assumed to be independent standard Wiener processes [s^1/2^]. *u*(*t*) is a feedback term [cm^2^/s^2^] modeling the coupling between the circulation strengths, the hydromechanical orientation mechanism, and the visual interaction of the fish with the walls, such that

$$\begin{array}{c} u(t)=\kappa \left(\Gamma_{r}\left(t\right)-\Gamma_{l}\left(t\right)\right)+u_{\text{h}}\left(t\right)+u_{\text{w}}\left(t\right). \end{array}$$ (3)

The feedback term *u*(*t*) acts differentially on Γ_*l*_(*t*) and Γ_*r*_(*t*), that is, it takes opposite signs in Eqs (2a) and (2b) to produce adequate turning maneuvers. For instance, when the fish performs clockwise turns, the vortex strengths should satisfy Γ_*l*_(*t*) > Γ_*r*_(*t*). Then, the feedback would tend to increase the circulation of the vortex on the left and decrease the circulation of the one on the right. The first term on the right hand side of Eq (3) corresponds to a classic bidirectional diffusive coupling, with *κ*[1/s] being the coupling strength [51, 52]. This positive parameter is associated with the ability of a fish to resume straight swimming after a maneuver. The diffusive coupling forces both processes to evolve along the synchronization manifold Γ_*l*_(*t*) = Γ_*r*_(*t*). The terms *u*_h_(*t*) and *u*_w_(*t*) capture the hydromechanical orientation mechanism and wall interactions through visual cues, respectively. Tactile interactions with the walls are separately addressed by modifying Eqs (2a) and (2b) to account for collisions.

### Hydromechanical feedback mechanism

**Fig 3:**
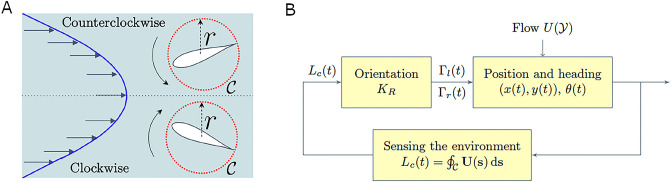
Hydromechanical feedback mechanism. (A) Example of rotation induced by a parabolic flow; the red circle of radius *r* is the approximation used for the fish perimeter in the computation of the local circulation of the background flow. (B) Block diagram describing the feedback mechanism to orient in the flow and perform rheotaxis.

Here, we model the feedback process allowing zebrafish to gather information from hydrodynamic cues and use them to orient in the flow, that is, modulating the vortex strengths through the term *u*_h_(*t*) in Eq (3). Similar to zebrafish larvae [25], we propose that adult zebrafish perform rheotaxis on the basis of an estimate of the local vorticity field. We compute the circulation of the background flow around a circle C with radius *r* centered at (*x*(*t*), *y*(*t*)), which approximates the fish perimeter (see Fig 3A),

$$\begin{array}{c} L_{c}\left(t\right)={∳}_{C}U(s)\;ds=-\pi r^{2}U^{\prime }\left(y\left(t\right)\right). \end{array}$$ (4)

Here, **U**(**s**) = [*U*(*s*_*y*_), 0] is the vector-field of the uni-axial background flow and the last equality is true up to the order $O(r^{4})$; see Materials and methods for details on the derivation. We set *r* = 1/2BL, with BL = 3.6 mm being the average fish body length. Positive values of *L*_*c*_(*t*) indicate that the background flow induces counterclockwise rotations, while negative values refer to clockwise rotations. The value of the local circulation *L*_*c*_(*t*) depends on the fish position in the swimming channel.

We consider the hydromechanical feedback *u*_*h*_(*t*) in Eq (3) to be a linear function of the local circulation of the background flow,

$$\begin{array}{c} u_{\text{h}}\left(t\right)=K_{R}\;K\left(t\right)\;L_{c}\left(t\right). \end{array}$$ (5)

Here, *K*_*R*_ [1/s] is a positive parameter weighting the hydrodynamic information, as illustrated in Fig 3B, and *K*(*t*) is a Boolean random variable. *K*(*t*) = 1 represents the case when the fish tracks the local circulation *L*_*c*_(*t*) to maneuver, while for *K*(*t*) = 0 this information is not utilized. The switching mechanism was introduced to model uncertainty in the rheotactic response, where fish alternates between time intervals following and ignoring the local circulation (see model calibration in Materials and methods for details on the estimation of *K*(*t*)). We model *K*(*t*) as a continuous-time Markov chain given by

$$\begin{array}{c} dK\left(t\right)=\left(1-K\left(t\right)\right)dN_{1}\left(t\right)-K\left(t\right)dN_{2}\left(t\right), \end{array}$$ (6)

where *N*_1_(*t*) and *N*_2_(*t*) are two independent counting processes whose increments *N*_1_(*t*′′) − *N*_1_(*t*′) and *N*_2_(*t*′′) − *N*_2_(*t*′) are Poisson random variables λ_1_(*t*′′ − *t*′), λ_2_(*t*′′ − *t*′) for any *t*′, *t*′′ ∈ *t* with *t*′′ > *t*′. Here, λ_1_ and λ_2_ are two positive parameters representing the rate of transitioning from not ignoring the local circulation to following it, and vice versa. For *K*(*t*) = 1, when *L*_*c*_(*t*) is positive, the fish feedback control mechanism would induce counterclockwise turns; in contrast, for negative values of *L*_*c*_(*t*) the turns would be clockwise (see Supporting information S1 Video).

### Wall interaction: Visual and tactile feedback

Here, we study the interaction of a fish with the walls, which comprises two different feedback mechanisms using vision and touch. Visual feedback is captured through *u*_w_ in Eq (3). Tactile feedback instead is modelled as a collision that modifies the evolution of the vortex strengths with respect to Eqs (2a) and (2b), as the fish collides with the walls.

Inspired by [34, 36], we quantified the wall effect by measuring the projected distance *d* and angle of collision *ϕ* which is measured from the wall axis to the projected heading vector (see Supporting information S2 Fig). We only considered those instances when the centroid was within 1 BL range from the wall.

The results indicate that a zebrafish rotates according to the sign of the angle *ϕ* when interacting with a wall (see Supporting information S2 Fig). Following [35], we model the visual feedback as a function of the projected distance and angle of collision which is given by

$$\begin{array}{c} u_{\text{w}}\left(t\right)=\frac{K_{W}}{Cd(t)+1}\text{sign}\left(\phi \left(t\right)\right), \end{array}$$ (7)

where *K*_*W*_ [cm^2^/s^2^] and *C* [1/cm] are positive constant parameters capturing the maximum intensity of turns and the decay of the wall effect as a function of the distance *d* [cm]. In the dark, we assume that animals do not have visual cues and this term is not present in the model, that is, *K*_*W*_ = 0.

To further delve into how fish interacts with the wall, we examined only instances when they were in close proximity or in direct contact to a wall. In these instances, the animal could exploit other sensing mechanisms beyond vision to avoid the wall. Our experimental results suggest that fish rotation in the vicinity of a wall depends on the sign of the angle *ϕ* (see Supporting information S2 Fig). We model the tactile component of turning in the vicinity of a wall, which is crucial for describing the wall interaction of the fish in the dark. In the vicinity of a wall, turns are captured through

$$\begin{array}{cc} \frac{d\Gamma_{l}\left(t\right)}{dt} & =\eta \;\text{sign}\left(\phi^{-}\left(t\right)\right),\;\;\;\;\;\text{for}\;\text{all}\;|x\left(t\right)|>x_{\text{max}}-\epsilon ,\;\left|y\left(t\right)\right|>y_{\text{max}}-\epsilon , \end{array}$$ (8a)

$$\begin{array}{cc} \frac{d\Gamma_{r}\left(t\right)}{dt} & =-\eta \;\text{sign}\left(\phi^{-}\left(t\right)\right),\;\text{for}\;\text{all}\;|x\left(t\right)|>x_{\text{max}}-\epsilon ,\;\left|y\left(t\right)\right|>y_{\text{max}}-\epsilon , \end{array}$$ (8b)

where *ϕ*^−^(*t*) denotes the angle of collision previous to the impact, *η* [cm^2^/s^2^] is the rate of turning once a collision occurs, and *ϵ* [cm] is an arbitrary small constant representing wall touching. We heuristically found that setting *η* = 10 cm^2^/s^2^ and *ϵ* = 0.001 cm reproduces realistic turns, as observed in real experiments.

There is an additional consideration to make for the right wall which corresponds to the test section outlet, shown in Fig 2. In this case, the fish experiences suction forces and could hit the wall while heading in a direction opposite to it, thereby preventing the use of Eqs (8a) and (8b) for capturing the impact. To account for this case and counter-balance the suction force, we should modify Eqs (8a) and (8b) as follows:

$$\begin{array}{cc} \frac{d\Gamma_{l}\left(t\right)}{dt} & =\eta ,\;\;\text{for}\;\text{all}\;x\left(t\right)>x_{\text{max}}-\epsilon ,\;\left|\theta \left(t\right)\right|>\frac{\pi }{2}, \end{array}$$ (9a)

$$\begin{array}{cc} \frac{d\Gamma_{r}\left(t\right)}{dt} & =\eta ,\;\;\text{for}\;\text{all}\;x\left(t\right)>x_{\text{max}}-\epsilon ,\;\left|\theta \left(t\right)\right|>\frac{\pi }{2}. \end{array}$$ (9b)

Here, the constraint on the heading angle guarantees that the animal is heading in the opposite direction to the right wall. Also, the signs in Eqs (9a) and (9b) are both positive, indicating that the interaction with this particular wall is repulsive to counter the suction force.

#### Model validation: Comparison between real and *in-silico* experiments

**Fig 4:**
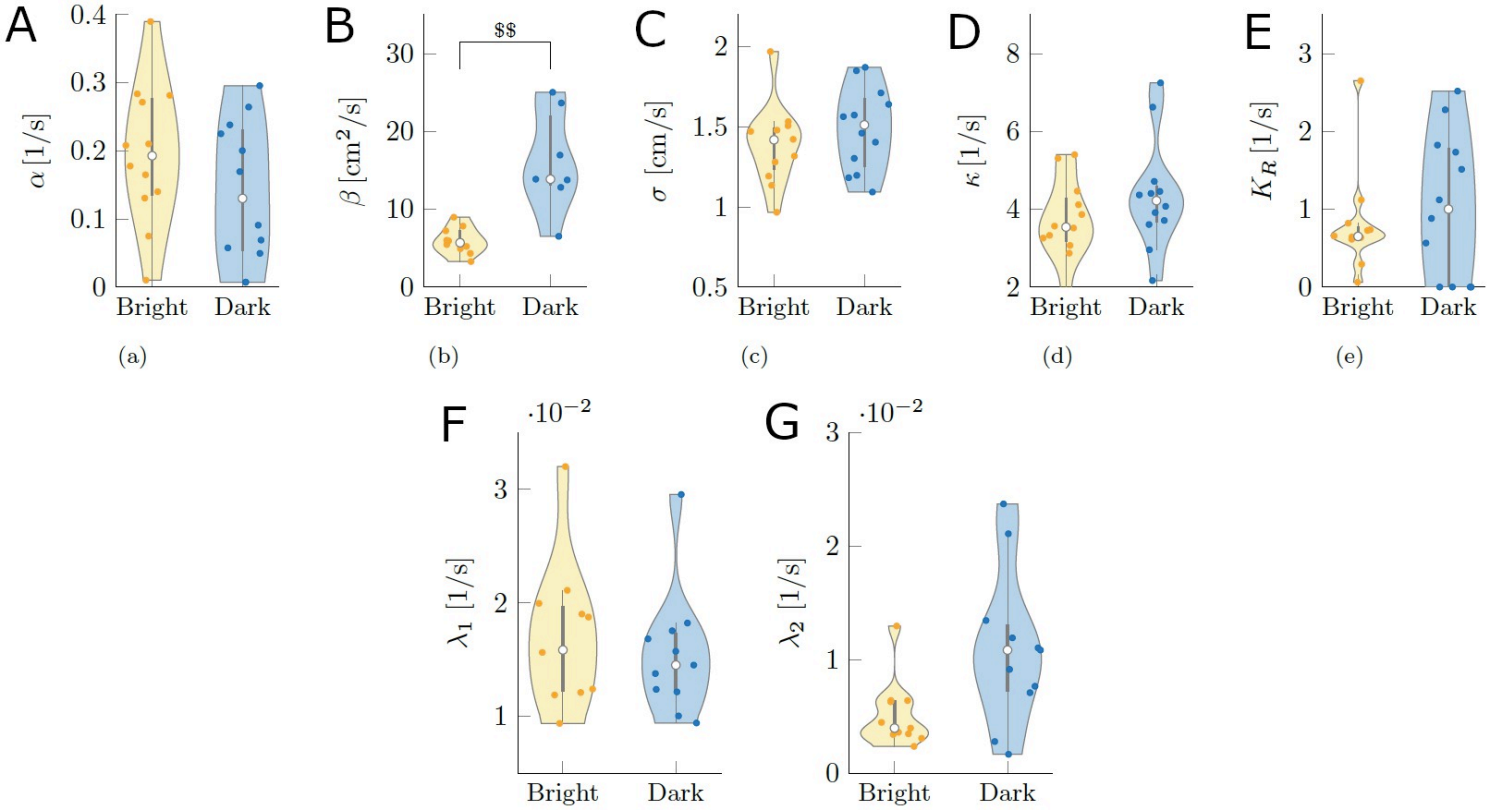
Calibrated model parameters for conditions Bright and Dark. (A) Linear rate of decay of the vortex strengths. (B) Baseline value of the vortex strengths. (C) Intensity of the noise added to the time-evolution of vortex strengths. (D) Coupling gain between vortex strengths associated with the ability of a fish to resume straight swimming after a maneuver. (E) Hydrodynamic feedback gain. (F-G) Rates of transitioning from dismissing to using information about the local circulation and vice versa. The gray bar in each violin plot details median (white dot), first and third quartiles, and lower and upper adjacent values. The colored area of a violin plot corresponds to the probability density of the data. Symbol $$ indicates a significant difference between conditions with *p* < 0.010.

We calibrated our model using experimental data, as detailed in Material and methods; the resulting parameter values are shown in Fig 4 for both conditions Bright and Dark. We found that the condition significantly influenced the baseline value of the circulation strengths *β* (*W* = 3;*p* < 0.010). We did not register a significant difference on the linear rate of decay of the vortex strengths *α*(*W* = 93;*p* = 1.000), the intensity of added noise *σ* (*W* = 56;*p* = 1.000), the coupling strength *κ* (*W* = 50;*p* = 1.000), hydrodynamic feedback gain *K*_*R*_ (*W* = 66;*p* = 1.000), λ_1_ (*W* = 72;*p* = 1.000), and λ_2_ (*W* = 27;*p* = 0.196).

**Fig 5:**
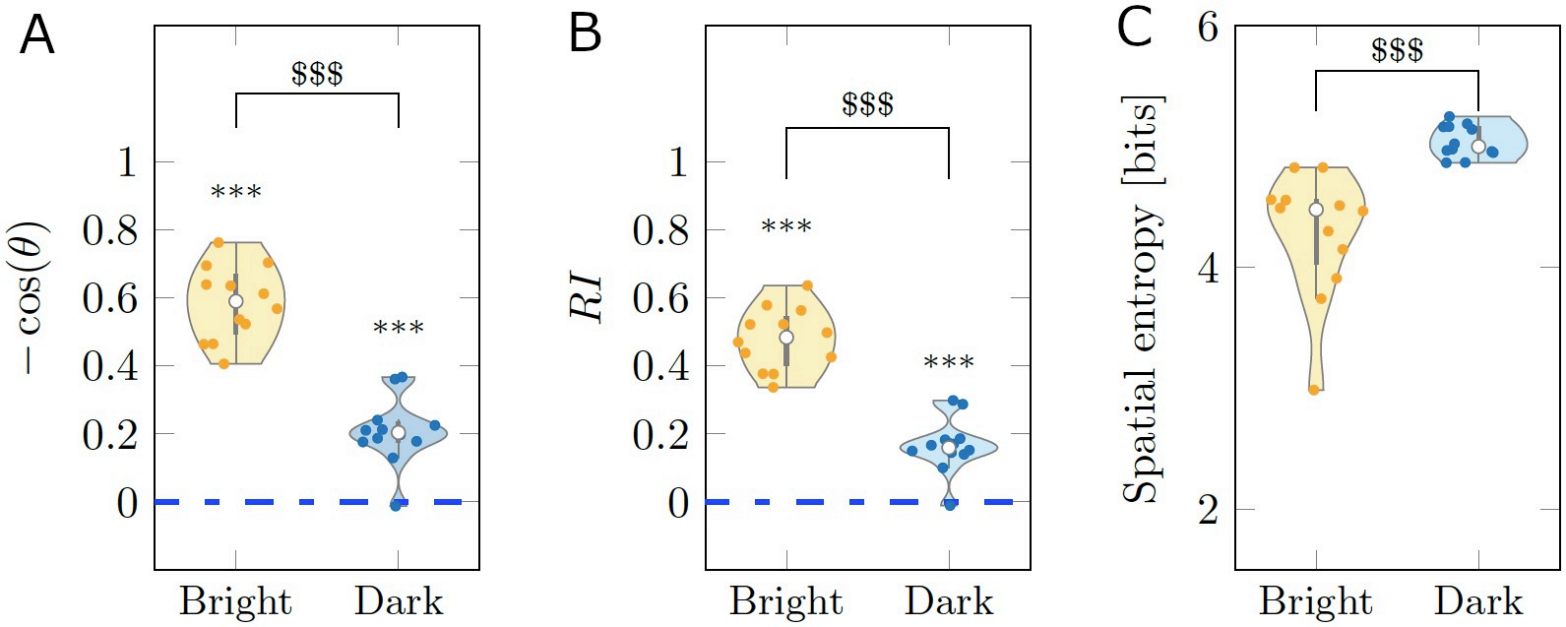
Average scoring of three behavioral metrics for *in-silico* experiments. (A-B) Rheotactic metrics taking values between −1 and 1 corresponding to biased headings downstream and upstream, respectively. (C) Spatial entropy, measuring the exploratory behavior across the test section. The average was taken over synthetic time series of 9, 000 points for each experimental subject. The gray bar in each violin plot details median (white dot), first and third quartiles, and lower and upper adjacent values. The colored area of a violin plot corresponds to the probability density of the data. Symbol $$$ indicate significant difference between conditions *p* < 0.010, respectively.

In order to validate the predictive power of our model, we conducted *in-silico* experiments consisting of 12 trials for each condition: Bright and Dark, as in the real experiment. *In-silico* experiments predicted relationships analogous to real experiments as shown in Fig 5. The cosine of the heading differed from chance in both Bright (*V* = 78;*p* < 0.001) and Dark (*V* = 77;*p* < 0.001). Similarly, *RI* registered significant differences in both Bright (*V* = 78;*p* < 0.001) and Dark (*V* = 77;*p* < 0.001). Pairwise comparisons between Bright and Dark indicated significant differences for the cosine of the heading (*W* = 144;*p* < 0.001), *RI* (*W* = 144;*p* < 0.001), and spatial entropy (*W* = 0;*p* < 0.001), while we only identify a weak trend for the variance of spatial entropy (*F* = 3.669;*p* = 0.068). Supporting information S2 Video and S3 Video show exemplary instances of rheotactic behavior predicted by the mathematical model in conditions Bright and Dark, respectively.

## Discussion

Rheotaxis is a complex multi-sensory process that involves the integration of different cues to orient in a flow and engage in counter-flow swimming. Toward a better understanding of how fish interacts with their surroundings and integrate different sensory cues during rheotaxis, we developed a data-driven mathematical model of zebrafish swimming in a flow. With respect to the state of knowledge on data-driven modeling of zebrafish locomotion, this study establishes the first mathematical model of swimming in a fluid flow. To generalize existing data-driven models that were intentionally developed for studying swimming in quiescent fluids [34–38], we tap into recent advancements in hydrodynamic modeling of fish swimming based on the finite-dipole paradigm [47, 48].

The proposed modeling framework is articulated in three main steps: (i) multi-sensing, through which the fish appraises its surroundings from visual, hydrodynamic, and tactile cues; (ii) orientation and navigation control, which uses the multi-sensory input to modulate the vortex strengths that are associated with self-propulsion; and (iii) motion in the flow based on the finite-dipole model, as a function of the background flow and the circulation strengths of the vortex pair. Our results indicate that hydromechanical feedback plays an important role in orientation and navigation whereby the fish tends to make turns by following the rotation induced by the flow, regardless of the availability of visual cues. This suggests that information about the environment provided by the lateral line alone could be sufficient to perform rheotaxis. This is also evident in our calibrated model parameters, where the feedback gain that is associated with hydromechanical sensory information did not vary with the illumination conditions. Our findings are in line with previous results in the literature, where it has been shown that the lateral line organ plays an important role in aiding the orientation of fish.

In a uniaxial flow, the feedback mechanism used by zebrafish reduces to tracking the gradient of the background flow. Specifically, the difference in the vortex strengths of the finite-dipole model is linearly controlled by the variation of the axial flow with respect to the width of the test section. Orientation strategies based on gradients have also been observed in other biological domains such as light gradient sensing in fish [55] where animals are able to track variations of light intensity and adjust their maneuvers [56]. Another example is chemical gradient sensing in cells [57, 58], where chemoattractant fields are sensed by proteins whose information is then used to modulate the orientation of the cell.

We observed that the scoring of behavioral metrics in real experiments was successfully paralleled by simulations. In particular, fish swimming in the dark displayed a higher locomotory activity in the test section, when compared to subjects in standard illumination conditions. Increased activity is likely related to an anxiety-related response, which is triggered by the presence of a dark, threatening environment, as widely documented in zebrafish literature on scototaxis [59]. *In-silico* experiments are also successful in predicting a significantly lower rheotactic performance for animals swimming in the dark. While sensing local circulation through the lateral line is not affected by the presence of visual cues, animal locomotion varies with the illumination conditions. Specifically, the mathematical model identifies that animals swimming in the dark have a higher relative speed with respect to the background flow than subjects in standard illumination conditions. This increased speed challenges the ability of zebrafish to adjust their orientation in response to the gradient of the background flow during rheotaxis.

Our approach has limitations that call for future research. First and foremost, the data-driven mathematical model focuses on two-dimensional swimming, thereby preventing the possibility of studying diving maneuvers along the height of the test section. Several studies [60–62] have pointed out the critical role of diving maneuvers on the response of this freshwater species, thereby suggesting the use of a three-dimensional ethogram for scoring zebrafish behavior. Three-dimensional effects are also likely to play a role on the difference between the rheotaxis metrics of real and *in-silico* experiments, whereby live animals have access to a richer flow physics than the two-dimensional background flow used in the simulations. Extending the proposed approach to three dimensions poses a number of methodological challenges, which requires a more complex representation than a vortex pair to encapsulate zebrafish swimming.

Second, we cannot exclude that zebrafish might exploit other sensory systems for performing rheotaxis. In our formulation, hydromechanical cues are the only source of rotational information for the fish, when swimming away from the wall. However, fish might integrate these cues with information of self-motion provided by the vestibular system [26, 27]. Disentangling the contribution of the vestibular system would require a different experimental set-up, possibly with zebrafish larvae. In fact, linear acceleration can be sensed by the semicircular canals, which are not functional at larval stages, allowing to hinder the effect of the vestibular system [25]. This approach could be used to study how rheotaxis changes across different stages and/or when senses are impaired, in order to differentiate individual contributions to this complex multi-sensory process.

Third, in more realistic environments, fish could exploit vortex wakes for maintaining upstream swimming via passive propulsion, by extracting energy from the background flow [63]. Hence, it is tenable that passive swimming could also contribute to fish rheotaxis, as evidenced for small scale systems such as human sperm [64] and artificial micro-swimmers [65]. Addressing these limitations calls for further research that combines experiments and mathematical models to better understand fish rheotaxis and uncover its underlying mechanisms.

In summary, we proposed a simple, yet effective, multi-sensory feedback control process for describing rheotaxis of an adult zebrafish. In particular, we incorporated three types of sensory feedback mechanism relying on visual, hydromechanical, and tactile cues. Interestingly, our model suggests that the gradient of the flow profile is the key information that drives rheotactic behavior. Similar to zebrafish larvae [25], our model indicates that rheotacting adults tend to follow the negative direction of the velocity gradient to adjust their orientation and swim upstream.

## Materials and methods

### Ethics statement

Experiments were performed in accordance with the guidelines and regulations approved by the University Animal Welfare Commitee (UAWC) of New York University under protocol number 13-1424.

### Animal care and maintenance

A total of 24 wild-type adult zebrafish (*Danio rerio*), 12 male and 12 female, were used in this study. The fish were purchased from Carolina Biological Supply Co. (Burlington, NC, USA), and housed in a 615 L vivarium tank divided into two compartments to mantain sexes separated. Fish were kept under a 12 h light/12 h dark photo-period and fed with commercial flake food once a day, approximately at 7 PM. Water parameters of the holding tanks were regularly checked, and temperature and pH were maintained at 26°C and 7.2, respectively. Prior to the beginning of the experiments, fish were acclimatized in the holding facility for one month.

### Experimental apparatus

**Fig 6:**
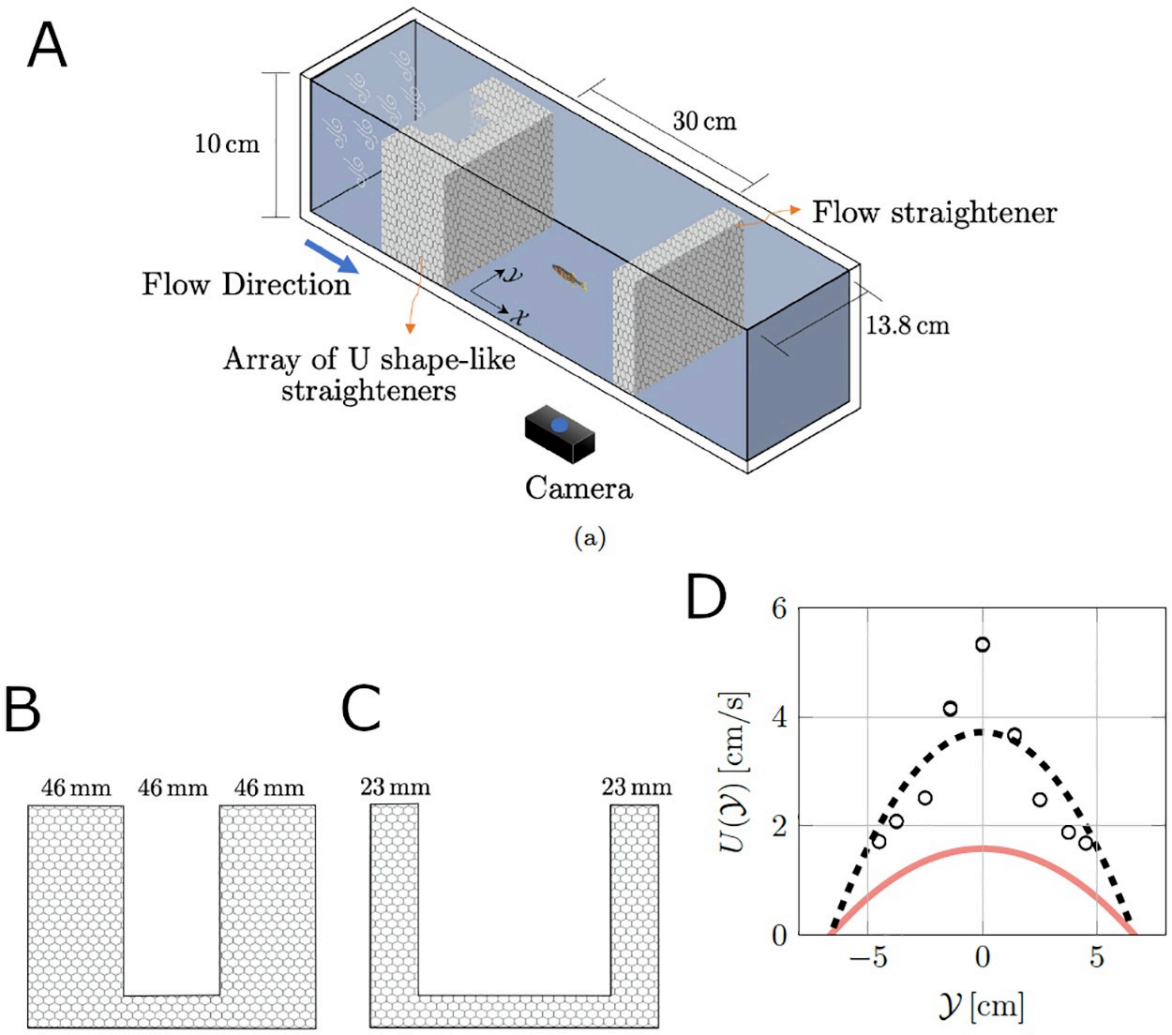
Experimental set-up. (A) Overview of the experimental apparatus. (B,C) U shape-like honeycomb grids for straightening the flow in the water channel. (D) Measurements of the flow velocity profile (black circles) and parabolic fit (black dash line) at the mid-span using laser Doppler velocimetry, along with the parabolic fit of flow profile from fish locomotion (solid red line).

The experimental set-up (see Fig 6A) consisted of a 151 L Blazka-type water channel (Engineering Laboratory Design Inc., Lake City, MI, USA), a video camera (Logitech C910 HD Pro Webcam without infrared filter, Logitech, Switzerland) located at the bottom of the channel, an array of lights, and black curtains to minimize outside visual stimuli. We used two different lighting systems for the Bright and Dark conditions. In particular, for the Bright condition, we used a pair of fluorescent lamps (Aqueon Full Spectrum Daylight T8, Aqueon, USA) located at the top of the channel along with a white plexiglass sheet to dim the light intensity and provide a homogeneous light background of 250 lx. For recording fish swimming in the dark, we used infrared lights (Iluminar IRC99 Series, Iluminar, Irvine, CA) with wavelength 940 nm, which is greater than the adult zebrafish threshold of spectral sensitivity [66]. Two pairs of infrared lights were located at the bottom and top of the water channel to provide a clear background for recording videos in the dark.

A test section of 30 cm × 13.8 cm (2*x*_max_ × 2*y*_max_) at a water height of 10 cm was arranged within the channel using flow straighteners, as shown in Fig 6A. The flow profile was created using an array of U-shaped flow straighteners with different opening sizes to manipulate the flow speed (see Fig 6B and 6C). We estimated the axial flow velocity utilizing the fish swimming trajectories for each experimental subject based on the following steps. First, we limited our analysis to instances when sin(*θ*(*t*)) ≠ 0, such that Eq (1) could be inverted to obtain $U\left(y\left(t\right)\right)=\frac{dx(t)}{dt}-\frac{dy(t)}{dt}\frac{\cos(\theta (t))}{\sin(\theta (t))}$. Second, we utilized a standard least squares method in Matlab (R2019b) to fit each data set with a parabola. By averaging the 24 fish, we determined a flow profile $U\left(Y\right)=-0.036Y^{2}+1.584$ (with units in cm and cm/s; see Fig 6D).

Interestingly, the advective velocity experienced by the fish was less than the velocity in the middle of the water height (5 cm from the bottom) through laser Doppler velocimeter (BSA, F50, Dantec, Denmark). Through velocimetry, we obtained five velocity measurements for nine different points across the test section (Y-coordinate). The fitted parabolic flow profile is shown in Fig 6D, for completeness. This discrepancy between identified data and velocimetry experiments is due to the fact that fish can swim along the *z*-axis, not only in the plane. We confirm this reduction along and across the tunnel by conducting finite element (FE) simulations of the fluid flow within the test section in the commercial FE software Comsol Multiphysics (see Supporting information S3 Fig).

### Experimental procedure

Two different illumination conditions were tested, namely Bright and Dark. Each trial consisted of three main phases. The first two phases were introduced for habituation to the new environment and the flow, while the third phase was the actual testing. Only the last phase was recorded. At the beginning of the trial, the animal was transferred from the vivarium to the water tunnel (using a hand net) and kept there for five minutes of habituation with the water velocity set to zero. Then, the water flow was turn on for two minutes of further habituation and five minutes of testing. A total of 24 naïve adult fish were tested, 12 (6 male and 6 female) for each condition (Bright and Dark).

### Tracking

A total of 300 s were recorded for each trial at 30 frames per second. All videos were post-processed using a foreground detection algorithm in Matlab (R2019b) for highlighting the animal shape on the image and improve the tracking process [67]. The resulting images were input to a slightly modified version of the multi-target tracking algorithm Peregrine [68], accounting for manual repairs in body shape tracking mode. The software fitted a parabola on the fish blob and returned: the fish centroid position (*x*(*t*), *y*(*t*)) with their respective velocities, shape parameters (coefficients of the parabola), and heading vector **h**(*t*) = [cos(*θ*(*t*)), sin(*θ*(*t*))], from which the heading angle and turn rate were calculated. For each experiment, we obtained time series of centroid coordinates, heading, and turn rate, consisting of 9, 000 samples corresponding to the total experimental time. All data can be found in the Supporting Information file S1 Data set.

### Statistical analyses and behavioral scoring

All statistical analyses were performed with the statistics software R (version 3.6.1). We used the Wilcoxon signed-rank test and the Mann-Whitney U test (Wilcoxon rank sum), with a significance level of 0.050, for comparing one-sample and two-sample data, respectively [69]. For testing the equality of two-sample data variances we use the Levene’s test [70] with a significance level of 0.050. To study rheotaxis, we averaged the time series of −cos(*θ*(*t*)) in each trial, and we scored *RI*, defined as the difference between the cumulative distribution functions of the absolute value of the heading and a uniform random variable [27]. More specifically, $RI=1-\left(2/\pi \right)\int_{0}^{\pi }\Lambda (|\theta |)\;d\theta$, with *Λ*(⋅) being the empirical cumulative distribution function. Here, *π*/2 represents the area under the curve of an empirical cumulative distribution function of a uniform random variable over the interval [0, *π*].

We further quantified the fish exploratory behavior in the test section through spatial entropy. This quantity was measured by first dividing the test section in 10 × 4 squares of approximately 3cm × 3.45cm each, corresponding to a grid of 1 BL in size. Then, using the centroid trajectory (*x*(*t*), *y*(*t*)), we estimated the probability of occupying each boxes in the grid, *p*_*i*_. The spatial entropy is then given by $-\sum_{i=1}^{40}p_{i}\;\log_{2}\left(p_{i}\right)$.

Comparisons of calibrated model parameters were conducted to elucidate the role of illumination thus reducing the number of model parameters and avoiding overfitting of the data. Comparisons were performed using seven independent Mann-Whitney U tests. Because hypotheses were not defined a priori on the parameter comparisons, we corrected for multiple comparisons using a Bonferroni adjusted significance level of 0.05/7 [71].

### *In-silico* experiments

We replicated the real experiment by considering 24 trials, 12 for Bright and 12 for Dark. We numerically integrated Eqs (1), (2), (3), (5) and (7) using the Euler-Maruyama scheme with a time step of 1/30 s [72], matching the sampling rate of the tracked data. To ensure convergence to a steady state probability distribution, we chose a simulation time of six times the experimental time (6 × 300 s), and we only considered the last 300 s. The parameter values *α*, *σ*, *κ*, *K*_*R*_, λ_1_, and λ_2_ were taken from Gaussian distributions corresponding to the data shown in Fig 4 across all 24 trials (Bright and Dark). Because the parameter *β* was significantly different between Bright and Dark, its value was drawn from two different Gaussian distributions corresponding to the data of each condition shown in Fig 4B. Given that the test section is rectangular, unrealistic turns or oscillations might arise on the corners due to their discontinuous nature [36]. To avoid this problem, we kept the angle to collision constant when the fish was inside a square region of 1cm^2^ on the corners.

### Derivation of the governing equations of the finite-dipole model

The zebrafish dipole representation is depicted in Fig 2. By adapting the equation set (2) from [48], the centroid position and heading angle can be obtained by integrating the following set of ODEs:

$$\begin{array}{c} \frac{dx(t)}{dt}=\frac{\Gamma_{l}\left(t\right)+\Gamma_{r}\left(t\right)}{4\pi l}\cos\left(\theta \left(t\right)\right)+\frac{U\left(y_{r}\left(t\right)\right)+U\left(y_{l}\left(t\right)\right)}{2}, \end{array}$$ (10a)

$$\begin{array}{c} \frac{dy(t)}{dt}=\frac{\Gamma_{l}\left(t\right)+\Gamma_{r}\left(t\right)}{4\pi l}\sin\left(\theta \left(t\right)\right), \end{array}$$ (10b)

$$\begin{array}{c} \frac{d\theta (t)}{dt}=\frac{U\left(y_{r}\left(t\right)\right)-U\left(y_{l}\left(t\right)\right)}{l}\cos\left(\theta \left(t\right)\right)+\frac{\Gamma_{l}\left(t\right)-\Gamma_{r}\left(t\right)}{2\pi l^{2}}, \end{array}$$ (10c)

where

$$\begin{array}{c} y_{l}\left(t\right)=y\left(t\right)+\frac{l}{2}\cos\left(\theta \left(t\right)\right),\;y_{r}\left(t\right)=y\left(t\right)-\frac{l}{2}\cos\left(\theta \left(t\right)\right). \end{array}$$ (11)

Considering that the animal thickness, *l* ∼ 5 mm, is small with respect to the dimensions of the water channel, we expand the velocity field at the location of the two vortices, *U*(*y*_*r*_(*t*)) and *U*(*y*_*l*_(*t*)), around the centroid coordinate *y*(*t*) using a Taylor series, yielding

$$\begin{array}{cc} U(y_{l}\left(t\right)) & =U\left(y\left(t\right)\right)+U^{\prime }\left(y\left(t\right)\right)\frac{l}{2}\cos\left(\theta \left(t\right)\right)+\frac{U^{\prime \prime }\left(y\left(t\right)\right)}{2}{\left(\frac{l}{2}\cos\left(\theta \left(t\right)\right)\right)}^{2}\\ & \;+\frac{U^{\prime \prime \prime }\left(y\left(t\right)\right)}{6}{\left(\frac{l}{2}\cos\left(\theta \left(t\right)\right)\right)}^{3}+O\left(l^{4}\right), \end{array}$$ (12a)

$$\begin{array}{cc} U(y_{r}\left(t\right)) & =U\left(y\left(t\right)\right)-U^{\prime }\left(y\left(t\right)\right)\frac{l}{2}\cos\left(\theta \left(t\right)\right)+\frac{U^{\prime \prime }\left(y\left(t\right)\right)}{2}{\left(\frac{l}{2}\cos\left(\theta \left(t\right)\right)\right)}^{2}\\ & \;-\frac{U^{\prime \prime \prime }\left(y\left(t\right)\right)}{6}{\left(\frac{l}{2}\cos\left(\theta \left(t\right)\right)\right)}^{3}+O\left(l^{4}\right), \end{array}$$ (12b)

where O(·) is Landau’s symbol. By considering a first order approximation in Eqs (12a) and (12b), we determine

$$\begin{array}{c} \frac{U\left(y_{r}\left(t\right)\right)+U\left(y_{l}\left(t\right)\right)}{2}≃U(y(t)), \end{array}$$ (13a)

$$\begin{array}{c} \frac{U\left(y_{r}\left(t\right)\right)-U\left(y_{l}\left(t\right)\right)}{l}≃-U^{\prime }\left(y\left(t\right)\right)\cos\left(\theta \left(t\right)\right). \end{array}$$ (13b)

Finally, replacing Eqs (13a) and (13b) in Eqs (10a) and (10b) yields Eqs (1a)–(1c).

### Estimation of the circulation strengths from experimental time series

To estimate the circulation strengths we used experimental data of the fish centroid position (*x*(*t*), *y*(*t*)), heading angle *θ*(*t*), and turn rate *ω*(*t*). Using a first order approximation, Eqs (10a) and (10b) can be written as

$$\begin{array}{c} \tilde{x}\left(kT\right)=\frac{T}{4\pi l}\left(\Gamma_{l}\left(kT\right)+\Gamma_{r}\left(kT\right)\right)\cos\left(\theta \left(kT\right)\right), \end{array}$$ (14a)

$$\begin{array}{c} \tilde{y}\left(kT\right)=\frac{T}{4\pi l}\left(\Gamma_{l}\left(kT\right)+\Gamma_{r}\left(kT\right)\right)\sin\left(\theta \left(kT\right)\right), \end{array}$$ (14b)

$$\begin{array}{c} \tilde{\omega }\left(kT\right)=\frac{1}{2\pi l^{2}}\left(\Gamma_{l}\left(kT\right)-\Gamma_{r}\left(kT\right)\right). \end{array}$$ (14c)

Here, *k* = 1, 2, …, *N* − 1 is the time step, *T* = 1/30 s is the video-camera sampling period, *N* = 9000 is the total number of samples, and

$$\begin{array}{c} \tilde{x}\left(kT\right)=x\left(\left(k+1\right)T\right)-x\left(kT\right)-\frac{(U\left(y_{r}\left(kT\right)\right)+U\left(y_{l}\left(kT\right)\right))T}{2}, \end{array}$$ (15a)

$$\begin{array}{c} \tilde{y}\left(kT\right)=y((k+1)T)-y(kT), \end{array}$$ (15b)

$$\begin{array}{c} \tilde{\omega }\left(kT\right)=\omega \left(kT\right)-\frac{U\left(y_{r}\left(kT\right)\right)-U\left(y_{l}\left(kT\right)\right)}{l}\cos\left(\theta \left(kT\right)\right), \end{array}$$ (15c)

with *U*(*y*_*r*_(*kT*)) and *U*(*y*_*l*_(*kT*)) being the flow velocities in correspondence of the right *y*_*r*_(*kT*) = *y*(*kT*) − (*l*/2)cos(*θ*(*kT*)) and left *y*_*l*_(*kT*) = *y*(*kT*) + (*l*/2)cos(*θ*(*kT*)) vortices, respectively.

By squaring both sides of (14a) and (14b), we determine that

$$\begin{array}{c} \sqrt{{\tilde{x}}^{2}\left(kT\right)+{\tilde{y}}^{2}\left(kT\right)}=\frac{T}{4\pi l}\left(\Gamma_{l}\left(kT\right)+\Gamma_{r}\left(kT\right)\right), \end{array}$$ (16)

Finally, from Eqs (14c) and (16) we obtain the sought expression of the circulations strengths as function of fish motion

$$\begin{array}{c} \Gamma_{l}\left(kT\right)=\pi l(\frac{2}{T}\sqrt{{\tilde{x}}^{2}\left(kT\right)+{\tilde{y}}^{2}\left(kT\right)}+l\tilde{\omega }\left(kT\right)), \end{array}$$ (17a)

$$\begin{array}{c} \Gamma_{r}\left(kT\right)=\pi l(\frac{2}{T}\sqrt{{\tilde{x}}^{2}\left(kT\right)+{\tilde{y}}^{2}\left(kT\right)}-l\tilde{\omega }\left(kT\right)). \end{array}$$ (17b)

### Expansion of the line integral for the local circulation

The fish perimeter is approximated by a circle C around the fish centroid (*x*(*t*), *y*(*t*)) defined by

$$\begin{array}{c} s_{x}=x\left(t\right)+r\cos\left(\varphi \right),\;s_{y}=y\left(t\right)+r\sin\left(\varphi \right),\;\text{for}\;\text{all}\;\varphi \in \left[0,2\pi \right]. \end{array}$$ (18)

The line integral in Eq (4) is thus given by

$$\begin{array}{c} {∳}_{C}U\left(s\right)\;ds=-r\int_{0}^{2\pi }U(y(t)+r\sin(\varphi ))\sin(\varphi )\;d\varphi . \end{array}$$ (19)

By a using a Taylor expansion of the velocity around *y*(*t*), we establish

$$\begin{array}{cc} {∳}_{C}U\left(s\right)\;ds & =-r\int_{0}^{2\pi }U(y(t))\sin(\varphi )\;d\varphi -r^{2}\int_{0}^{2\pi }U^{\prime }\left(y\left(t\right)\right)\sin^{2}\left(\varphi \right)\;d\varphi \\ & -\frac{r^{3}}{2}\int_{0}^{2\pi }U^{\prime \prime }\left(y\left(t\right)\right)\sin^{3}\left(\varphi \right)\;d\varphi +O\left(r^{4}\right). \end{array}$$ (20)

Finally, from the fact that $\int_{0}^{2\pi }\sin(\varphi )\;d\varphi =0$, $\int_{0}^{2\pi }\sin^{2}\left(\varphi \right)\;d\varphi =\pi$, and $\int_{0}^{2\pi }\sin^{3}\left(\varphi \right)\;d\varphi =0$ we derive Eq (4).

### Model calibration

We began by approximating the solutions of the stochastic differential equations in Eqs (2a) and (2b) away from the wall (neglecting *u*_w_), using the Euler-Maruyama method, thereby yielding the following Markov chain:

$$\begin{array}{cc} \Gamma_{l}\left(\left(k+1\right)T\right) & =\Gamma_{l}\left(kT\right)+[\alpha \left(\beta -\Gamma_{l}\left(kT\right)\right)+\kappa \left(\Gamma_{r}\left(kT\right)-\Gamma_{l}\left(kT\right)\right)+K\left(kT\right)K_{R}L_{c}\left(kT\right)]T\\ & +\sigma \sqrt{\Gamma_{l}\left(kT\right)T}\xi_{l}\left(kT\right), \end{array}$$ (21a)

$$\begin{array}{cc} \Gamma_{r}\left(\left(k+1\right)T\right) & =\Gamma_{r}\left(kT\right)+[\alpha \left(\beta -\Gamma_{r}\left(kT\right)\right)+\kappa \left(\Gamma_{l}\left(kT\right)-\Gamma_{r}\left(kT\right)\right)-K\left(kT\right)K_{R}L_{c}\left(kT\right)]T\\ & +\sigma \sqrt{\Gamma_{r}\left(kT\right)T}\xi_{r}\left(kT\right), \end{array}$$ (21b)

where *ξ*_*l*_ and *ξ*_*r*_ are two independent standard Gaussian random variables, with zero mean and unit variance. *K*(*kT*) is the experimental Boolean random variable taking value 1 if the fish follows the circulation *L*_*c*_(*kT*) and 0 otherwise.

To estimate *K*(*kT*), we quantified the level of synchronization between the turn rate of the fish *ω*_*a*_(*kT*) = Γ_*l*_(*kT*) − Γ_*r*_(*kT*) and *L*_*c*_(*kT*). We first normalized both time-series on the interval [−1, 1] by dividing each of them by the maximum absolute value over the entire time span yielding ${\hat{\omega }}_{a}\left(kT\right)$ and ${\hat{L}}_{c}\left(kT\right)$. Next, we smoothed ${\hat{\omega }}_{a}\left(kT\right)$ utilizing a moving average filter in Matlab (R2019b) to attenuate noise. Then, we assessed synchronization by defining the synchronization error $e\left(kT\right)=\sqrt{{\left({\hat{\omega }}_{a}\left(kT\right)-s\left(kT\right)\right)}^{2}+{\left({\hat{L}}_{c}\left(kT\right)-s\left(kT\right)\right)}^{2}}$ with $s\left(kT\right)=({\hat{\omega }}_{a}\left(kT\right)+{\hat{L}}_{c}\left(kT\right))/2$ being the average signal. By manually inspecting all 24 trials, we found that *e*(*kT*)<0.35 over a time window greater than 2 s corresponded to instances in which the fish tracked the local circulation, that is, it performed a complete turning maneuver following the rotation indicated by the circulation. Hence, we imposed *K*(*kT*) = 1 if *e*(*kT*)<0.35 for all the frames within any 2s-window containing instant *kT*. The use of a continuous window allows for mitigating the effect of chance, whereby the fish may inadvertently follow the circulation during its swimming, despite not using it for decision-making.

After some algebraic manipulations, Eqs (21a) and (21b) can be rewritten as

$$\begin{array}{c} Z_{l}\left(kT\right)≔f\left(\Gamma_{l}\left(kT\right),\Gamma_{l}\left(\left(k+1\right)T\right),\Gamma_{r}\left(kT\right),\alpha ,\beta ,\sigma ,\kappa ,K_{R}\right)=\sqrt{{\left(\frac{\sigma }{K_{0}}\right)}^{2}T}\xi_{l}\left(kT\right), \end{array}$$ (22)

$$\begin{array}{c} Z_{r}\left(kT\right)≔f\left(\Gamma_{r}\left(kT\right),\Gamma_{r}\left(\left(k+1\right)T\right),\Gamma_{l}\left(kT\right),\alpha ,\beta ,\sigma ,\kappa ,K_{R}\right)=\sqrt{{\left(\frac{\sigma }{K_{0}}\right)}^{2}T}\xi_{r}\left(kT\right), \end{array}$$ (23)

where the scalar function *f* (*X*, *Y*, *Z*, *α*, *β*, *σ*, *κ*, *K*_*R*_) is given by

$$\begin{array}{c} f\left(X,Y,Z,\alpha ,\beta ,\sigma ,\kappa ,K_{R}\right)=\frac{Y+X\left(\alpha T+\kappa T-1\right)-\alpha \beta T-\kappa TZ-K\left(kT\right)K_{R}L_{c}\left(kT\right)}{K_{0}\sqrt{X}}, \end{array}$$ (24)

with *K*_0_ being an arbitrary positive constant, introduced to avoid numerical issues when the circulations strengths are close to zero. To calibrate the model we estimated the parameters **Θ** = [*α*, *β*, *σ*/*K*_0_, *κ*, *K*_*R*_] using the maximum likelihood estimation method [73] by solving the following constrained optimization problem:

$$\begin{array}{c} \hat{\Theta }=\text{arg}\;\min_{\Theta }-[\sum_{k=1}^{N^{*}}\log g(\Theta ,Z_{l}\left(kT\right))+\log g(\Theta ,Z_{r}\left(kT\right))] \end{array}$$ (25a)

$$\begin{array}{cc} & \text{such}\;\text{that}\;\sigma^{2}<2\alpha \beta , \end{array}$$ (25b)

where N* < *N* is the total number of samples where the fish was swimming away from the wall. The function *g*(**Θ**, *Z*) is the Gaussian distribution with zero mean and variance (*σ*/*K*_0_)^2^ *T*, given by

$$\begin{array}{c} g\left(\Theta ,Z\right)=\frac{1}{\sqrt{2\pi T{\left(\frac{\sigma }{K_{0}}\right)}^{2}}}\;e^{-\frac{Z^{2}}{2T{\left(\frac{\sigma }{K_{0}}\right)}^{2}}}. \end{array}$$ (26)

Next, to estimate the parameters λ_1_ and λ_2_ of the continuous-time Markov chain in (6), we counted the number of transitions of *K*(*kT*) from ignoring the circulation to following it and vice versa. The estimated parameters are shown in Fig 4 for the 24 experimental trials.

**Fig 7:**
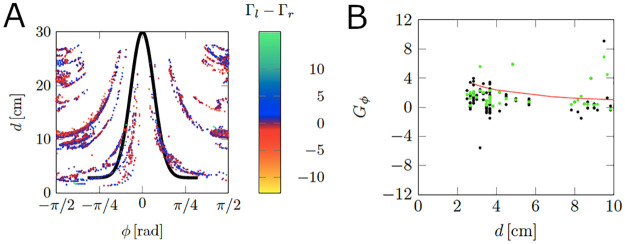
Illustration of the wall calibration process. (A) Two-dimensional projection of the difference of vortex strengths Γ_*l*_ − Γ_*r*_, as a function of the projected distance, *d*, and angle to collision, *φ*, for one trial from Bright. The black curve is a normal function utilized to select relevant values of (*φ*, *d*) associated with those instances when the fish turns according to the angle to collision *ϕ*. (B) Example of calibration of the wall function. Black dots correspond to *G*_*ϕ*_ and green dots correspond to the filtered output of |*G*_*ϕ*_|. The red line is the fitted wall function.

Moreover, for calibrating the wall parameters in Eq (7), we implemented the following steps:

(i) We first extracted instances when the fish turns according to the opposite sign of the angle to collision *ϕ*, that is, blue points (Γ_*l*_ − Γ_*r*_ > 0) for *ϕ* < 0 and red points (Γ_*l*_ − Γ_*r*_ < 0) for *ϕ* > 0 as shown in Fig 7A. To undertake this step, we utilized a cutoff function, which was informed by the following rationale. As the angle *ϕ* approaches ±*π*/2 or the distance to collision *d* increases, fish turns becomes less predictable. Hence, we retained pairs (*ϕ*, *d*) such that |*g*_*ϕ*_(*ϕ*) − *d*|<*δ* and |*ϕ*|<*ϕ*_0_, where *ϕ*_0_ and *δ* are cutoff parameters and $g_{\phi }\left(\phi \right)=a_{g}+b_{g}\;e^{-{\left(\phi /c_{g}\right)}^{2}}$ (Black curve in 7a). By manually examining the 12 trials in Bright, we found that setting *ϕ*_0_ = 1, *a*_*g*_ = 2.8 cm, *b*_*g*_ = 27.2 cm, *c*_*g*_ = 0.26, and *δ* = 1 was a valid choice to extract all relevant maneuvers.
(ii) To understand how fish turn based on the vicinity to a wall, we defined *G*_*ϕ*_ as the quantity collecting the values of the difference of circulation strengths (Γ_*l*_ − Γ_*r*_), corresponding to the points (*ϕ*, *d*) obtained from the previous step. For the example shown in Fig 7A, the points *G*_*ϕ*_ correspond to black dots. Next, we used a non-parametric locally weighted least squares (LOESS) filter in Matlab (R2019b) with a 5% span on the absolute value of *G*_*ϕ*_ to smoothen the data. The results are the green dots shown in Fig 7B;
(iii) The output of the LOESS filter, *y*_*d*_, was utilized as input to fit the wall function *K*_*W*_/(*Cy*_*d*_ + 1) using the nonlinear least-squares solver of Matlab (R2019b). The fitted function corresponds to the red curve in Fig 7B and
(iv) Because we used the difference of circulation strengths for the fitting, the estimate of *K*_*W*_ should be corrected to obtain the true amplitude of turns corresponding to each circulation strengths. Hence, we computed the maximum value of Γ_*l*_ and Γ_*r*_ across all time instances near a wall. *K*_*W*_ was selected as the maximum between the values obtained in (iii) and (iv). Results are reported in Table 1.

**Table 1:** Calibrated wall parameters for the 12 fish tested in standard illumination (condition Bright). For experiments in the dark, *K*_*W*_ is set to zero and this form of interaction is absent.

Trial	*K*_*W*_ [1/s]	*C* [cm]
1	23.727	-
2	48.112	2.172
3	63.173	2.237
4	23.170	-
5	29.646	2.195
6	78.167	2.196
7	21.005	-
8	33.066	-
9	32.606	2.194
10	39.661	2.502
11	33.658	2.158
12	32.696	-
Mean	38.224	2.236
Median	32.881	2.195

We remark that our model is calibrated by splitting our data set into two: (i) data of fish interacting with the wall, and (ii) data of fish swimming away from walls. We set 1 BL of distance from the wall as a threshold to split the data set. This separation provides enough data points for the condition of fish swimming away from the wall, needed to guarantee convergence of the optimization problem in Eq (25). This distance, however, could be within the capabilities of zebrafish to detect walls [74]. We verified that the calibrated parameters values do not change considerably by slightly increasing this threshold to 1.2 BL (∼4 cm) and 1.4 BL (∼5 cm).

## Supporting information

S1 Data set (ZIP)

S1 Fig Example of vortex strenghts estimated from real data. Example of estimated vortex strengths Γ_*l*_ and Γ_*r*_ from real data of two different subjects in conditions Bright and Dark. (A,B) Histograms and (C,D) phase plots of vortex strengths for conditions (A,C) Bright and (B,D) Dark. In both cases, the distributions of Γ_*l*_ and Γ_*r*_ are highly correlated with *R*^2^ values of 0.848 and 0.779, for Bright and Dark, respectively.
(TIFF)

S2 Fig Analysis of the wall interaction. (A) Illustration of the process to compute the projected distance and angle to collision. We have that *ϕ* = *π*/2 if the fish is heading straight to the wall, and *ϕ* = 0 if it is perfectly aligned to the wall axis. In addition, *ϕ* > 0 (clockwise) and *ϕ* < 0 (counterclockwise) indicate instances when a fish approaches the wall with its right or left side, respectively. (B) Quantification of zebrafish tendency to make turns based on the angle *ϕ*. We scored *F*_*ϕ*_ as the percent of instances when the sign of turn rate *ω*(*t*) was the opposite of the sign of *ϕ*, irrespective of the distance from it. The blue dashed-line represents the random chance level of 50%. We compared the value of *F*_*ϕ*_ for conditions Bright and Dark with chance. We registered a significant difference for condition Bright (*V* = 75;*p* < 0.010) while we fail to register a significant difference for Dark (*V* = 54;*p* = 0.067). (C) Quantification of the ability of fish to turn away from a wall for distances to collision less than 1 BL. We document a significant difference for both conditions Bright (*V* = 65;*p* < 0.010) and Dark (*V* = 62;*p* < 0.010), this observation offers partial support in favor of the presence of other mechanisms to detect walls when swimming in close proximity. The gray bar in each violin plot details median (white dot), first and third quartiles, and lower and upper adjacent values. The colored area of a violin plot corresponds to the probability density of the data. Symbol ** indicates a significant difference from chance with *p* < 0.010.
(TIF)

S3 FigSimulation of the flow profile.We considered a 30 × 10 × 13.8 cm (length × height × width) parallelepiped, in which we solve static, incompressible, laminar Navier-Stokes equations through the “Laminar Flow” environment in COMSOL Multiphysics. We set non-slip wall conditions on the bottom, left, and right boundaries, and an open boundary on the top surface, where no viscous stress is generated. Between the inlet and outlet, we imposed a pressure difference. For all our simulations, we used COMSOL built-in water material properties and a “Normal” size mesh. We first estimated the required pressure difference to generate the maximum experimental fluid speed at the center of the section, assuming a Poiseuille flow within a circular pipe, with a diameter equal to the hydraulic diameter of the rectangular section, yielding 0.038 Pa. By imposing this pressure, we obtained a larger speed for all the points but the central one. To identify the correct value of the pressure drop to reconstruct the experimental profile, we carried out a parametric analysis in which we varied the pressure drop between 0.0021 Pa and 0.038 Pa in 20 steps. We find that the sum of squared errors of fluid speed at the measurement positions is minimized for a pressure difference of 0.0172 Pa. From these simulations, we conclude that the average fluid speed within the section is not equal to the maximum velocity within the X-Y plane passing through the center of the test section, that is, the plane in which fluid speed was experimentally measured. By computing the average speed on the plane parallel to Y-Z passing through the section of the test section, we obtain a value of 2.3 cm/s, which is approximately 70% of the maximum speed at the center of the section, 3.2 cm/s.(TIF)

S1 Video Illustrative example of zebrafish turning maneuvers according to the local circulation. Panels on the left illustrate the real experiment (top) and tracking (bottom). The right upper panel illustrates how the turn rate is adjusted according to the local circulation of the background fluid flow. The evolution of heading angle is shown in the bottom left panel.
(M4V)

S2 Video Example of rheotaxis predicted by the proposed mathematical model in condition Bright. (MOV)

S3 Video Example of rheotaxis predicted by the proposed mathematical model in condition Dark. (MOV)
