# Correction: Modeling multi-sensory feedback control of zebrafish in a flow

**DOI:** 10.1371/journal.pcbi.1010222

**Published:** 2022-06-02

**Authors:** Daniel A. Burbano-L., Maurizio Porfiri

## Notice of republication

This article was republished on May 9^th^, 2022, to correct a calibration error affecting data reported in Table 1, Figs. 1, 4, 5, 6, 7, S1 and S2, and S1 Data set. This error does not have consequences for the validity of the model, nor the statistical analysis in the original submission. Please download the article again to view the correct version. The originally published, uncorrected article and the republished, corrected article are provided here for reference.

## Supporting information

S1 File Originally published, uncorrected article. (PDF)

S2 File Republished, corrected article. (PDF)
