# Supplementary material for: Modeling multi-sensory feedback control of zebrafish in a flow
Source: PLoS Comput Biol. 2021 Jan 22;17(1):e1008644. doi: 10.1371/journal.pcbi.1008644 (PMC7857640; doi:10.1371/journal.pcbi.1008644)
Supplement: S1 Data set — (ZIP) [file pcbi.1008644.s001.zip › Readme.rtf]

Manuscript: Modeling multi-sensory feedback control of zebrafish in a flowby Daniel A. Burbano-L. and Maurizio PorfiriThere are two attached files; namely, Data_Bright.dat and Data_Dark.datcorresponding to the experimental data of the two conditionsin which fish swam with normal illumination and in the dark.Each file has five columns:- Column 1:    the first column is the fish ID (from 1 to 24 animals, 12 for condition Bright and 12 different animals for condition Dark)- Column 2,3: the second and third columns correspond to the centroid position x [cm], y[cm], respectively.- Column: 4:   Heading angle [rad], Angles are between -pi to pi. 0 indicates heading to the right, pi/2: up, -pi/2: down, pi/-pi: left (see Figure 1 in the manuscript)- Column 5:    Turn rate [rad/s][-] For each fish ID, there are 9000 samples corresponding to the 300s (5 min) of recorded experiments.[-] the sampling rate is 1/30 s, (30 is the frame rate)Questions? Please contact Prof. Maurizio Porfiri: mporfiri@nyu.edu
