# Supplementary figures and images for: Modeling multi-sensory feedback control of zebrafish in a flow
Source: PLoS Comput Biol. 2021 Jan 22;17(1):e1008644. doi: 10.1371/journal.pcbi.1008644 (PMC7857640; doi:10.1371/journal.pcbi.1008644)

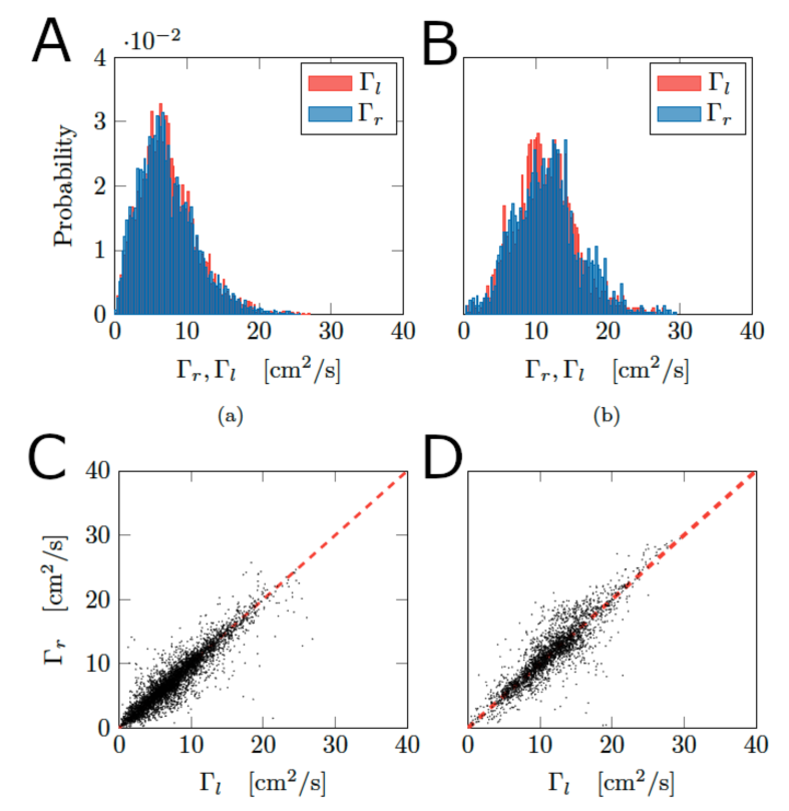

Supplement: S1 Fig — Example of estimated vortex strengths Γl and Γr from real data of two different subjects in conditions Bright and Dark. (A,B) Histograms and (C,D) phase plots of vortex strengths for conditions (A,C) Bright and (B,D) Dark. In both cases, the distributions of Γl and Γr are highly correlated with R2 values of 0.848 and 0.779, for Bright and Dark, respectively. (TIFF) [file pcbi.1008644.s002.tiff]

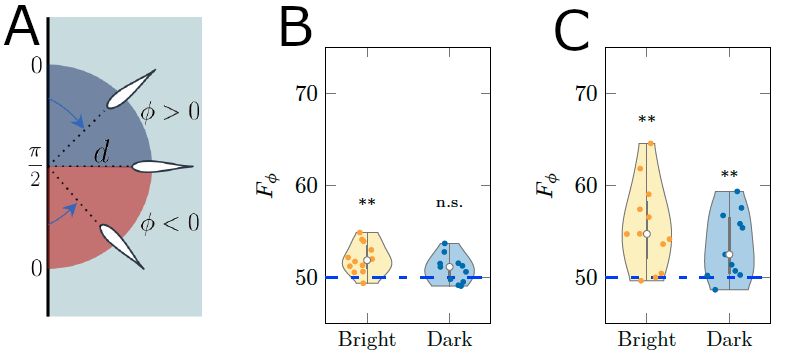

Supplement: S2 Fig — (A) Illustration of the process to compute the projected distance and angle to collision. We have that ϕ = π/2 if the fish is heading straight to the wall, and ϕ = 0 if it is perfectly aligned to the wall axis. In addition, ϕ > 0 (clockwise) and ϕ < 0 (counterclockwise) indicate instances when a fish approaches the wall with its right or left side, respectively. (B) Quantification of zebrafish tendency to make turns based on the angle ϕ. We scored Fϕ as the percent of instances when the sign of turn rate ω(t) was the opposite of the sign of ϕ, irrespective of the distance from it. The blue dashed-line represents the random chance level of 50%. We compared the value of Fϕ for conditions Bright and Dark with chance. We registered a significant difference for condition Bright (V = 75;p < 0.010) while we fail to register a significant difference for Dark (V = 54;p = 0.067). (C) Quantification of the ability of fish to turn away from a wall for distances to collision less than 1 BL. We document a significant difference for both conditions Bright (V = 65;p < 0.010) and Dark (V = 62;p < 0.010), this observation offers partial support in favor of the presence of other mechanisms to detect walls when swimming in close proximity. The gray bar in each violin plot details median (white dot), first and third quartiles, and lower and upper adjacent values. The colored area of a violin plot corresponds to the probability density of the data. Symbol ** indicates a significant difference from chance with p < 0.010. (TIF) [file pcbi.1008644.s003.tiff]

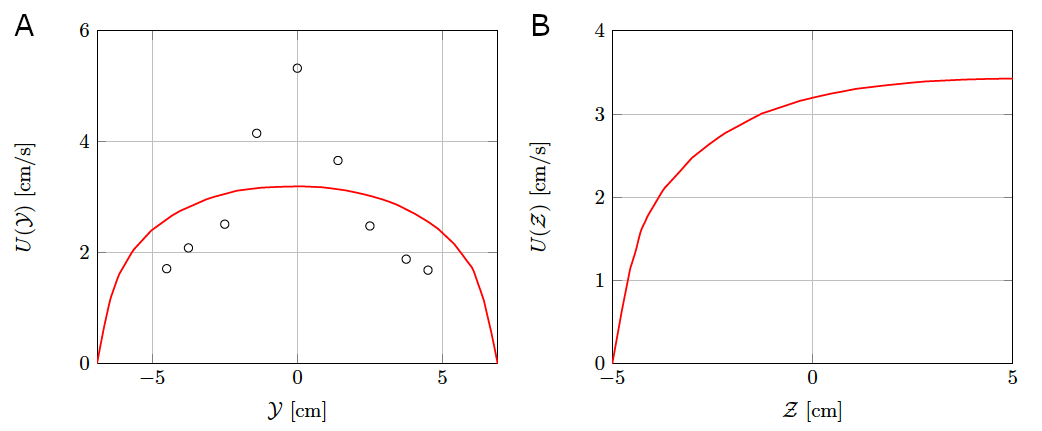

Supplement: S3 Fig — We considered a 30 × 10 × 13.8 cm (length × height × width) parallelepiped, in which we solve static, incompressible, laminar Navier-Stokes equations through the “Laminar Flow” environment in COMSOL Multiphysics. We set non-slip wall conditions on the bottom, left, and right boundaries, and an open boundary on the top surface, where no viscous stress is generated. Between the inlet and outlet, we imposed a pressure difference. For all our simulations, we used COMSOL built-in water material properties and a “Normal” size mesh. We first estimated the required pressure difference to generate the maximum experimental fluid speed at the center of the section, assuming a Poiseuille flow within a circular pipe, with a diameter equal to the hydraulic diameter of the rectangular section, yielding 0.038 Pa. By imposing this pressure, we obtained a larger speed for all the points but the central one. To identify the correct value of the pressure drop to reconstruct the experimental profile, we carried out a parametric analysis in which we varied the pressure drop between 0.0021 Pa and 0.038 Pa in 20 steps. We find that the sum of squared errors of fluid speed at the measurement positions is minimized for a pressure difference of 0.0172 Pa. From these simulations, we conclude that the average fluid speed within the section is not equal to the maximum velocity within the X-Y plane passing through the center of the test section, that is, the plane in which fluid speed was experimentally measured. By computing the average speed on the plane parallel to Y-Z passing through the section of the test section, we obtain a value of 2.3 cm/s, which is approximately 70% of the maximum speed at the center of the section, 3.2 cm/s. (TIF) [file pcbi.1008644.s004.tif]
